# The Influence of Tacrolimus Exposure and Metabolism on the Outcomes of Kidney Transplants

**DOI:** 10.3390/biomedicines12051125

**Published:** 2024-05-18

**Authors:** Rima Maslauskiene, Ruta Vaiciuniene, Aurelija Radzeviciene, Peteris Tretjakovs, Gita Gersone, Edgaras Stankevicius, Inga Arune Bumblyte

**Affiliations:** 1 Department of Nephrology, Medical Academy, Lithuanian University of Health Sciences, LT-44307 Kaunas, Lithuania; ruta.vaiciuniene@lsmu.lt (R.V.); ingaarune.bumblyte@lsmu.lt (I.A.B.); 2 Institute of Physiology and Pharmacology, Medical Academy, Lithuanian University of Health Sciences, LT-44307 Kaunas, Lithuania; aurelija.radzeviciene@lsmu.lt (A.R.); edgaras.stankevicius@lsmu.lt (E.S.); 3 Department of Human Physiology and Biochemistry, Riga Stradins University, LV-1007 Riga, Latvia; peteris.tretjakovs@rsu.lv (P.T.); gita.gersone@rsu.lv (G.G.)

**Keywords:** kidney transplantation, tacrolimus monitoring, C/D ratio, tacrolimus coefficient of variation, neutrophil gelatinase-associated lipocalin, kidney injury molecule-1

## Abstract

Tacrolimus (TAC) has a narrow therapeutic window and patient-specific pharmacokinetic variability. In our study, we analyzed the association between TAC exposure, metabolism, and kidney graft outcomes (function, rejection, and histological lesions). TAC trough (C_0_), coefficient of variation (TAC CV), concentration/dose ratio (C/D), and biomarkers related to kidney injury molecule-1 (KIM-1) and neutrophil gelatinase lipocalin (NGAL) were analyzed. We examined 174 patients who were subjected to a triple immunosuppressive regimen and underwent kidney transplantation between 2017 and 2022. Surveillance biopsies were performed at the time of kidney implantation and at three and twelve months after transplantation. We classified patients based on their Tac C/D ratios, classifying them as fast (C/D ratio < 1.05 ng/mL × 1/mg) or slow (C/D ratio ≥ 1.05 ng/mL × 1/mg) metabolizers. TAC exposure/metabolism did not significantly correlate with interstitial fibrosis/tubular atrophy (IF/TA) progression during the first year after kidney transplantation. TAC CV third tertile was associated with a higher chronicity score at one-year biopsy. TAC C/D ratio at three months and Tac C_0_ at six months were associated with rejection during the first year after transplantation. A fast TAC metabolism at six months was associated with reduced kidney graft function one year (OR: 2.141, 95% CI: 1.044–4.389, *p* = 0.038) and two years after transplantation (OR: 4.654, 95% CI: 1.197–18.097, *p* = 0.026), and TAC CV was associated with reduced eGFR at three years. uNGAL correlated with IF/TA and chronicity scores at three months and negatively correlated with TAC C_0_ and C/D at three months and one year. Conclusion: Calculating the C/D ratio at three and six months after transplantation may help to identify patients at risk of suffering acute rejection and deterioration of graft function.

## 1. Introduction

The most frequently prescribed maintenance immunosuppressive regimen following renal transplantation includes mycophenolate mofetil (MMF), tacrolimus (TAC), and steroids. According to the most recent data from the Scientific Registry of Transplant Recipients (SRTR), over 90% of transplant recipients in the USA receive TAC as part of their treatment [1]. While TAC is highly effective in preventing rejection, it has some significant limitations. These include a narrow therapeutic window and pharmacokinetic variability between patients, and even for the same patient over time. Personalized medicine aims to achieve tailored immunosuppression management to enhance patient and graft survival after kidney transplantation. Given the delicate balance between alloimmune risk and TAC toxicity, the monitoring of the effects of TACs has received considerable attention. Most transplant centers routinely apply therapeutic drug monitoring (TDM) utilizing tacrolimus through concentration measurements. While C_0_ whole-blood trough concentrations do not definitely correlate with clinical outcomes [2]*,* several studies analyzed other markers of tacrolimus exposure or metabolism, including tacrolimus concentration dose ratio (C/D) [3,4,5,6], the coefficient of variation for tacrolimus (TAC CV) [7,8], the tacrolimus daily dose concentration ratio [9], time in the therapeutic range [10,11], area under the concentration–time curve [12,13], and intra-cellular TAC concentrations in peripheral blood mononuclear cells and graft issue [14,15]. There is currently no agreement on the most accurate and suitable additional therapeutic-drug-monitoring method for clinical practice.

The long-term outcomes of renal grafts can be influenced by inadequate or excessive immunosuppression, both of which can lead to poor outcomes such as graft failure, rejection, infectious complications, toxicity, and malignancies. Chronic allograft dysfunction is the leading cause of long-term graft loss [16]. Surveillance biopsies performed at different time intervals can identify the presence of subclinical inflammation and the progression of renal scarring. Several early studies found that lower tacrolimus trough levels are associated with the progression of interstitial fibrosis and tubular atrophy (IF/TA) [17]; other studies found an association between TAC dose reduction and subclinical inflammation but not IF/TA progression [18,19] and the risk of the appearance of donor-specific antibodies [19]. Higher intra-patient variability in TAC C_0_ has also been associated with a faster progression of IF/TA [20,21], worse graft survival, and a higher risk of rejection [22].

For the non-invasive prediction of acute and chronic CNI-induced nephrotoxicity due to induced kidney vasoconstriction and interstitial fibrosis, kidney damage markers, including neutrophil gelatinase-associated lipocalin (NGAL) and kidney injury molecule-1 (KIM-1), have been studied and may have value [23,24].

This study aims to analyze the association of tacrolimus exposure and metabolism markers with kidney graft histological lesions, kidney damage biomarkers, and function, and evaluate which measurement method is more appropriate for clinical practice. The novelty of this study lies in the fact that we not only analyzed the association between different measurements of tacrolimus, such as TAC C_0_*,* TAC C_0_ CV, and C/D ratio, with respect to kidney graft function over three years, but also evaluated their association with the biomarkers KIM-1 and NGAL and *g*raft histological lesions monitored using zero-time and surveillance biopsies.

## 2. Materials and Methods

### 2.1. Study Design and Patients

**Figure 1:**
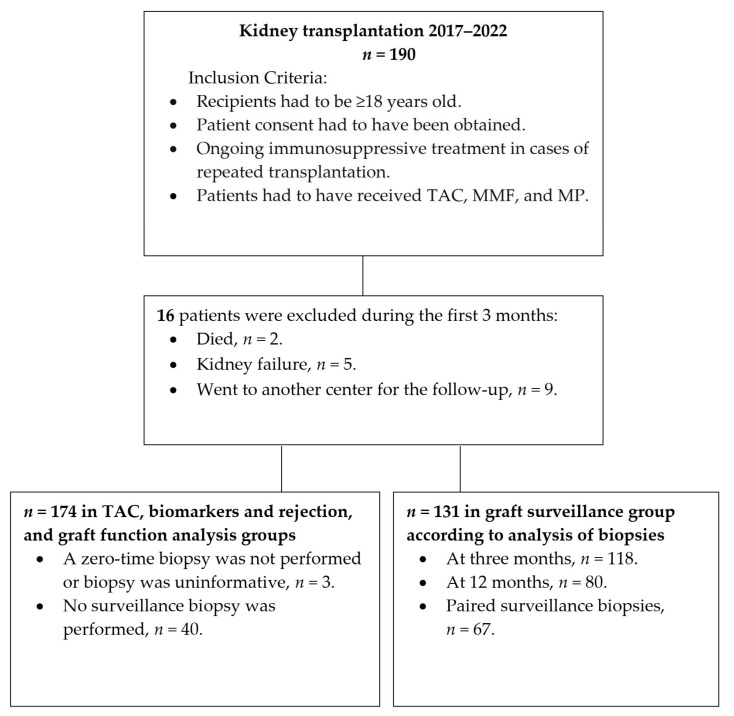
Study flow chart.

This prospective observational study included kidney recipients who received transplants at the Kaunas Clinics of the Lithuanian University of Health Sciences Hospital from May 2017 to December 2022. All kidney transplant donors and recipients were of Caucasian ethnicity. The recipients’ follow-up data from three years after transplantation were analyzed. The observation period ended early in cases of graft failure, recipient death, or loss to follow-up. This study included adult patients treated with prolonged-release tacrolimus, mycophenolate mofetil (MMF), (standard protocol: 2 g per day first 3 months after transplantation and 1 g per day later), or enteric-coated mycophenolic acid (EC-MPA) and methylprednisolone (MP) during the follow-up. To assess the association between tacrolimus and histological changes, a cohort of patients who underwent both zero-time and at least one protocol biopsy was analyzed. The patients were divided into two groups according to IF/TA progression. Through a multivariate analysis to identify the factors of IF/TA progression, rejection, and graft function, we compared not only measures of tacrolimus exposure/metabolism but also several essential characteristics of the donors and recipients. To evaluate the relationship between TAC and graft function, separate TAC measurements were analyzed using eGFR <60 mL/min/1.73 m^2^ vs. ≥60 mL/min/1.73 m^2^. A total of 174 kidney recipients were included (Figure 1).

All clinical data were obtained from medical records in accordance with the standard of care and routine clinical practice. Laboratory values were analyzed in the hospital’s laboratory as part of routine follow-up care. Kidney function was evaluated monthly from three to twelve months after transplantation. Serum creatinine concentrations were measured using the kinetic Jaffe (compensated) method, which is traceable to the isotope dilution mass spectrometry (IDMS) reference method (Analyzer AU680, Beckman Coulter, Brea, CA, USA). The estimated glomerular filtration rate (eGFR) was ascertained using the Chronic Kidney Disease Epidemiology Collaboration (CKD-EPI, 2012) equation. Expanded criteria donors (ECD) were categorized according to the United Network for Organ Sharing criteria [25]. Delayed graft function (DGF) was defined as the requirement for dialysis during the first week after transplantation.

### 2.2. Biomarker Measurement

Blood and urine samples for biomarker measurements were collected at the same time as the kidney biopsies, while patients were hospitalized for kidney biopsy procedures at three months and one year after transplantation. Two commercially available biomarkers for kidney injury were tested: NGAL in serum and urine (enzyme-linked immunosorbent assay (ELISA) kit (Human Lipocalin-2/NGAL ELISA Kit; Sigma-Aldrich Chemie GmbH, St. Louis, MI, USA)) and urine KIM-1 (Quantikine ELISA kit (R&D Systems Europe, Ltd.; Abingdon, UK). Serum and urine samples were analyzed in Riga Stradins University’s Department of Human Physiology and Biochemistry Laboratory as recommended by the manufacturer. A detailed description of the biomarker analysis process is described in a previously published article [26].

### 2.3. Histological Analysis

The urologist performed a time-zero graft biopsy in the kidney transplant operating room during surgery when kidney blood flow was restored. According to our transplant center protocol, surveillance biopsies were performed at three and twelve months after kidney transplantation under ultrasound guidance by trained radiologists using a 16-gauge automated needle. The pathologists at the National Center of Pathology evaluated the histological findings of the kidney biopsies according to the updated Banff Classification (1997) [27]. Interstitial inflammation (i) was defined as an i-score ≥ 1, while biopsies without interstitial infiltrates (i-score = 0) were classified as no inflammation. The chronicity score was defined as the sum of glomerular basement membrane double contours (cg), interstitial fibrosis (ci), tubular atrophy (ct), vascular fibrous intimal thickening (cv), mesangial matrix expansion (mm), and arteriolar hyalinosis (ah) [28]. IF/TA score (ci + ct) was calculated for each biopsy, and the progression of IF/TA between biopsies was defined as the difference in ci + ct score from the previous surveillance biopsy ≥1 (on a scale of 0 to 6). In this study, patients were divided into an IFTA progression group (for which the ci+ct scores in surveillance biopsies were higher than those for the zero-time biopsy) and a stable group (for which there was no difference between the biopsies’ ci+ct scores).

### 2.4. Immunosuppression and Tacrolimus Monitoring

Recipients were divided into immunological risk groups based on pre-transplant panel reactive antibody (PRA), HLA mismatch, and the presence of donor-specific antibodies (DSA). Low immunological risk was assessed for non-sensitized patients with 0–2 HLA mismatches. Patients were assessed as high immunological risk when PRA of 50% or more was detected, or PRA 10–49% with three or more HLA mismatches, repeated transplantation, or who were positive for DSA. Other patients were assigned to the medium-risk immunological group.

Standard immunosuppression included induction therapy with basiliximab before kidney transplantation and four days after transplantation for kidney recipients with medium or high immunological risk. High-risk recipients with a calculated PRA of 50% and more, or recipients for whom DSA were detected, received rabbit anti-thymocyte globulin. After the surgery, the patients were treated with triple immunosuppression therapy consisting of prolonged-release tacrolimus, mycophenolate mofetil (MMF) or equimolar doses of enteric-coated mycophenolic acid (EC-MPA), and steroids. The target tacrolimus concentration was 7–15 ng/mL during the first 3 months after transplantation and 5–10 ng/mL between 3 months and one year after transplantation. Routinely, all patients received MMF 2 g/d (or EC-MPA 0.72 g bid) during the first three months, and this treatment was then tapered to 1.0 g/d. Tacrolimus concentrations were determined using the affinity chromium immunoassay method via a Dimension EXL 200 (Siemens) Integrated Chemistry System. Tacrolimus levels were tested monthly for three months and after one year. Additional samples were taken when the patients arrived for the biopsy procedure. The patients’ Tac C_0_ data were manually reviewed, excluding values affected by untimely testing, treatment of infection, rejection, or other reasons. At least three Tac C_0_ measurements for an individual patient had to be available for IPV calculation. The intra-patient variability (IPV) in tacrolimus trough levels was evaluated as the coefficient of variability (CV), calculated as follows: CV (%) = (SD/mean) ∗ 100. For the present study, we considered analyzing the CV of TAC C_0_ between six and twelve months after transplantation. According to previous studies, we considered the highest upper tertile of CV to correspond to a high risk of adverse clinical outcomes. We employed the tacrolimus concentration/dose ratio (C/D) to analyze TAC metabolism at 3, 6, and 12 months and two and three years. According to previous studies, a C/D ratio < 1.05 µg/L × 1/mg was considered suggestive of a fast tacrolimus metabolism, and a C/D ≥ 1.05 µg/L × 1/mg was considered suggestive of a slow metabolism [3].

### 2.5. Statistical Analysis

Descriptive statistics were presented as means (SD) for normally distributed data, and for continuous variables not following a normal distribution, the median (interquartile range or the minimum and maximum values) was used. Categorical variables were reported as frequencies (percentages). The Chi-squared test was employed for categorical data analysis, and the McNemar test was applied to related nominal data. The Student’s *t*-test was utilized to compare normally distributed continuous variables. Differences were assessed using the Mann–Whitney U-test and the Kruskal–Wallis test for continuous variables with skewed distributions. Non-normally distributed biomarker values were log-transformed. Depending on the distribution of variables, correlations between continuous variables were assessed using Pearson or Spearman tests. A logistic regression analysis was performed to analyze the associations between histological lesions, graft function, and clinical data. Factors that were significantly different between the groups according to the univariate analyses were included in multivariate analyses. The results were presented as odds ratios (OR) with a 95% confidence interval (95% CI) and the *p*-value of a likelihood-ratio test. All tests of significance were two-sided, with *p* < 0.05 considered significant. Data were analyzed using IBM SPSS Statistics for Windows, Version 29.0.2.0 (Armonk, NY, USA: IBM Corp).

## 3. Results

### 3.1. Characteristics of the Study Population

The demographic and transplant-related variables of the studied cohort are summarized in Table 1. Half of the donors (50.0%) met the extended donor (ECD) criteria. In total, 87.9% of the patients were on hemodialysis, 4% were preemptive, and 8% were on peritoneal dialysis before transplantation. A total of 90.5% of the recipients were undergoing their first kidney transplantation, while 7.5% had undergone repeated transplantations. The most common causes of end-stage renal disease were chronic glomerulonephritis (39.7%), polycystic kidney disease (12.6%), and diabetes (10.3%). During the follow-up period, biopsy-proven acute rejection episodes were diagnosed for 30 (17.4%) recipients; 24 (13.8%) cases occurred during the first year after transplantation and 11 (6.3%) of them during the first month. Cytomegalovirus infection was treated in 24 cases (13.8%), and polyoma virus BK infection was treated in 7 cases (4%). During post-transplant follow-up, post-transplant diabetes mellitus occurred in 28 (16.1%) cases. The percentage of individuals with fast tacrolimus metabolism decreased over time: 43.6% of the cohort were considered fast metabolizers at three months, changing to 42.6% at six months, 32.9% at one year, 22.7% at two years, and 18.8% at three years.

According to the protocol, starting from the third month, tacrolimus doses were reduced and significantly differed from the values at one year (8.0 (2–25) vs. 6 (1–20); *p* <0.001), and TAC-C0 was correspondingly lower at a later period (9.34 ± 2.93 vs. 7.5 ± 2.4 vs.; *p* = 0.014). IPV ranged from 6.08 to 59.77*%* (26.78 ± 11.25). The highest tertile of the TAC variability coefficient was ≥30.6251% (mean, 39.5 ± 7.42; median, 37.03; min, 30.63%; and max, 59.77%).

### 3.2. Graft Biopsy Group Analysis

In the zero-time biopsy, there were 17 cases (13%) with IF/TA (*ci* ≥1 and/or *ct* ≥1) that were mild *(*8.4*%*) and moderate (4.6%). IF/TA score increased over time when comparing biopsies at three months and one year (1.14± 1.22 (0–6) vs. 0,3 ± 5.12 (0–2); *p* = 0.02). In the three-month biopsies, there were 44 cases (37.9%) with mild (19%), moderate (16.4%), and severe (2.6%) IF/TA. In the surveillance biopsies at one year, 48 cases (60.8%) of IF/TA had mild (24.1%), moderate (27.8%), and severe (8.9%) lesions. The degree of inflammatory lesions did not significantly change between biopsies (*p* = 0.375), although tubulitis incidence tended to be higher in the one-year biopsies (8.6 vs. 23.1; *p* = 0.118). Global glomerulosclerosis percentage (4.87 ± 7.43 vs. 4.40 ± 7.95, *p* = 0.625) and arteriolar hyalinosis (*p* = 0.625) did not differ between three- and one-year biopsies; however, chronicity score (1.46 ± 1.57; (min 0, max 6) vs. 2.18 ± 1.96 (min 0, max 9), *p* < 0.001) significantly increased.

In the graft biopsies group, acute rejection episodes were diagnosed for twenty-one recipients during the first year after kidney transplantation; eleven cases occurred during the first month.

Overall progression of IF/TA was observed in 55% of patients (72 cases): from the zero-time biopsy to three months in 36.2% of biopsies, and from 3 months to 1 year in 46% of cases. IF/TA progression was associated with recipient age, donor hypertension, rejection episodes, and the presence of interstitial inflammation (i) (*p* = 0.02) and inflammation with tubulitis (“i + t”) (*p* = 0.013) at a one-year biopsy. There were no significant differences between biopsies in the arteriolar hyalinosis score, serving as a potential marker for tacrolimus nephrotoxicity (Supplement Table S1). Table 2 shows the properties of TAC exposure and metabolism stratified to IF/TA progression in the survival biopsies.

No statistically significant association was found between Tac C0 concentration, the C/D ratio, the TAC IPV, and IF/TA progression. Also, no significant association was found between the periods from the zero-time biopsy to the 3-month biopsy and from the 3-month to 1-year biopsies (Supplement Table S2). TAC C_0_ and C/D ratio values at six months negatively correlated with IF/TA score for the one-year biopsy (r = −0.320, *p* = 0.004 and r = −0.269, *p* = 0.001) but did not correlate with IFTA progression. Fast TAC metabolizer status was not significantly associated with IF/TA progression. There was no significant association between the TAC CV third tertile and IF/TA progression, but it was associated with a higher chronicity score at the one-year biopsy (OR: 1.429, 95% CI: 1.092–1.869, *p* = 0.009).

sNGAL was tested at the same time as a surveillance biopsy was performed and correlated with inflammation at the biopsy at three months (i) (r = 0.317, *p* = 0.006) and inflammation with tubulitis (i + t) (r = 0.314, *p* = 0.006); uNGAL correlated with IF/TA score (r = 0.303, *p*= 0.046) and chronicity score (r = 0.347, *p* = 0.021), but these relationships were not significant at the one-year biopsy. Only sNGAL at three months was associated with IF/TA progression in one-year surveillance biopsy (OR: 0.149*,* 95% CI: 0.027–0.815, *p* = 0.028). No significant associations were found between urinary NGAL and KIM-1 and the progression of IF/TA (Supplement Table S2).

### 3.3. Association between Tacrolimus Monitoring and Biomarkers

uNGAL negatively correlated with TAC C_0_ at three months (r = −0.283, *p* = 0.025) and at one year (r = −0.585, *p* = 0.05), and with C/D ratio at three months (r = −0.464, *p* = 0.034) and at one year (r = −0.525, *p* = 0.015). Fast metabolizers at six months correlated with uNGAL at one year (r = 0.567, *p* = 0.007). C/D ratio at six months correlated with uKIM-1 (r = −0.412, *p* = 0.041).

### 3.4. Association between Tacrolimus Monitoring and Rejection

Most of the rejection episodes were diagnosed during the early period after kidney transplantation, so the relationship between tacrolimus exposure and metabolism and first-year rejections was analyzed. Based on a multivariable logistic regression analysis model, after adjusting variables deemed significant in univariate analysis, it was found that the TAC-C/D ratio at three months and Tac C_0_ at six months, DGF, and the presence of diabetes mellitus were associated with rejection during the first year after transplantation Table 3.

There was no significant association between TAC CV (*p* = 0.765) or TAC CV third tertile (*p* = 0.514) and rejection during the first year.

There was no significant association between biomarkers and rejection frequency or later graft function.

### 3.5. Tacrolimus Measurements and Graft Function

Regardless of metabolizer status, no significant differences were detected between TAC metabolizer type, body mass index, age, and gender.

Logistic regression was conducted to evaluate tacrolimus measurements’ association with reduced graft function at one year, two years, and three years after transplantation. The patients were divided into two groups based on their eGFR levels: eGFR ≥ 60 mL/min/1.73 m^2^, and <60 mL/min/1.73 m^2^.

Univariable logistic regression analyses showed that TAC C_0_ (OR: 0.84, 95% CI: 0.725–0.972, *p* = 0.019) and fast metabolizer type at six months (OR: 2.141 95% CI: 1.044–4.389, *p* = 0.038), along with several clinical factors, namely, donor age, donor hypertension, duration of renal replacement therapy before transplantation, cold ischemia time, usage of angiotensin-converting enzyme (ACE) inhibitors at one year after transplantation, and IF/TA progression in surveillance biopsies, were significantly associated with 1-year eGFR. However, in multivariate analysis, only donor and recipient clinical data, constituting untested TAC monitoring parameters, were significantly associated with graft function.

Univariate analyses of graft function at two years revealed that TAC C_0_ and fast metabolizer type at six months and several clinical factors, namely, donor age, affliction with hypertension, donor evaluation, rejection during the first year*,* higher IF/TA, and chronicity scores for one-year surveillance biopsy, were significantly associated with two-year eGFR < 60 mL/min/1.73 m^2^. In multivariate logistic regression, being of fast TAC metabolizer type at six months remained a significant graft function predictor, along with donor age and chronicity score Table 4.

In an analysis of graft function at three years, from among all the tested tacrolimus-monitoring methods, only TAC CV was a significant factor *(*OR: 1.068, 95% CI: 1.011–1.129, *p* = 0.019), along with the donors’ factors and chronic histological lesions. Although statistically insignificant, the TAC CV’s highest tertile was associated with reduced eGFR *<* 60 mL/min/1.73 m^2^ at three years *(p =* 0.051).

## 4. Discussion

This prospective study analyzed the association between several therapeutic drug-monitoring methods for tacrolimus exposure and metabolism, such as TAC C_0_, TAC C_0_ CV, C/D ratio, the biomarkers KIM-1 and NGAL, IF/TA progression in kidney grafts in zero-time and surveillance biopsies, kidney graft rejection, and later kidney function over a three-year period. For eighty percent of the patient biopsies, proven rejection occurred during the first year after transplantation. A lower C/D ratio at three months and lower Tac C_0_ at six months were associated with rejection. It was found that TAC metabolizer status, especially in the first year after transplantation, may help to predict graft function at one year and two years.

No significant association was found between TAC C_0_ concentration, the C/D ratio, TAC IPV, the highest tertile of TAC CV, and IF/TA progression in surveillance biopsies, but the TAC CV’s highest tertile was associated with a higher chronicity score at one-year biopsy, which is an independent risk factor of graft failure [28]. uNGAL negatively correlated with TAC C_0_ and C/D ratio at three months and one year, and with IF/TA and chronicity scores at three-month biopsies; therefore, this may be taken to be a possible marker of CNI-induced renal injury. sNGAL at three months was associated with IF/TA progression in surveillance biopsies. The biomarkers KIM-1 and NGAL were not found to be significantly associated with rejection or eGFR over the follow-up period.

Numerous studies have analyzed the relationship between TAC IPV and patient and graft survival; however, few studies have focused on chronic histological lesion analyses [22]. Several authors have analyzed the dynamics of IF/TA inception through subsequent protocol biopsies, using different TAC C_0_ CV levels of tertiles ranging from 22.1 to 44.2% [10,21,29]. Studies analyzing the association between tacrolimus and IF/TA progression have obtained conflicting results. In the study by Vanhove et al., paired surveillance biopsies were performed at 3 months and 2 years after transplantation. The patients with the third IPV tertile had an increased risk of developing moderate to severe fibrosis and tubular atrophy within two years post-transplantation compared to the lowest tertile. The authors found that kidney function did not deteriorate during the 2-year follow-up period; this indicates that high IPV may be associated with the accelerated progression of chronic histologic lesions before any evidence of kidney dysfunction. [21]. We found no relation between TAC CV and IFTA progression, but an association with a higher chronicity score was obtained. In this study, only 8.9% of the IF/TA group biopsies found lesions that were determined to be severe, with most biopsies finding minor or moderate lesions. On the other hand, 1-year protocol biopsies may not be sufficient to assess IFTA progression. It is possible that changes associated with tacrolimus metabolism and exposure might be observed in future protocol biopsies at 2 and 3 years.

A recent study by Kim H et al. showed that their low-IPV group had better five-year graft survival rates than the high-IPV group, and high IPV was linked to CNI nephrotoxicity [29]. This study data showed that higher TAC CV was associated with reduced graft function not in the early period but at three years after transplantation. Another large study by Kim EJ found that high TAC IPV significantly increases the risk of graft failure and antibody-mediated rejection in patients at high immunological risk. However, among the low-immunological-risk group patients, death-censored graft survival and rejection were not significantly different in terms of TAC IPV [7]. These results could also explain the findings regarding IPV in this study, as only 12.8 percent of the recipients were at high immunological risk.

The usefulness of NGAL and KIM-1 measurements in kidney transplantations has mainly been assessed in urine or blood samples taken early—i.e., hours or days—after transplantation. The value of biomarker measurements taken later in the period after transplantation remains less investigated. A few studies have analyzed NGAL and KIM-1 at a later period after kidney transplantation. In animal models, NGAL produced by macrophages plays a critical role in renal fibrosis [30]. In a non-transplant patient study, plasma NGAL (pNGAL) levels correlated with the degree of interstitial cell infiltration and fibrosis [31]. A recent study found that higher uNGAL levels at 3, 6, 9, and 12 months were associated with a higher kidney graft fibrosis score [32]. In early studies, during a later period after transplantation, uKIM-1 was associated with the presence of IF/TA [33] and negatively correlated with graft function [34]. In this study, serum NGAL was tested at three months when surveillance biopsies were performed, and it was associated with inflammation. At three months after transplantation, uNGAL showed a negative correlation with IF/TA and chronicity scores. While serum NGAL was associated with the progression of IF/TA at the time of the surveillance biopsy at three months, it did not have such an association at one year. However, it is essential to note that the small sample of biomarkers used in this study may have influenced the results. An early study that monitored urinary KIM-1 over a median six-year post-transplant period found that this is an independent predictor of long-term graft loss [35]. Another recent study by Kremer et al. analyzed the relationship between pNGAL measured at a median age of 5.4 years in stable kidney transplant recipients and kidney graft outcomes. pNGAL was found to be strongly associated with graft failure, especially among patients with preexisting poor kidney function and proteinuria. Elevated pNGAL concentrations were strongly associated with an increased risk of death-censored graft failure [36]. We did not find a significant association between biomarkers and graft function at three months and over a one-year period.

This study revealed a negative correlation between uNGAL and TAC C_0_ and C/D at three months and at one year. uKIM-1 at six months correlated with C/D ratio, and uNGAL at one year correlated with fast metabolizer status at six months. A possible explanation for these findings is that biomarkers such as NGAL and KIM-1 may be associated with CNI-induced renal vasoconstriction [23,24].

The findings of the TAC C/D ratio and graft outcome analysis are not homogenous. Some authors found that high tacrolimus clearance was significantly associated with the development of IF/TA in the first year following renal transplantation [9]. A recent study that tested whether TAC C/D was associated with rejection during the first year, graft, and patient survival at one, three, six, nine, and twelve months does not support tailoring tacrolimus monitoring therapy based on C/D in the early post-transplant period [37]. Other authors found that fast metabolizers, classified as such according to C/D ratio measurement at three months, showed a faster decline in eGFR within five years after transplantation and a higher rejection rate than slow metabolizers [3]. In this study, the TAC C_0_ at six months and the C/D ratio at three months were associated with rejection, and being a fast metabolizer at three and six months was linked to reduced graft function. This study confirms the results of other studies on the prognostic importance of TAC concentration measured after six months for renal outcomes after transplantation. The results can be attributed to immunosuppression typically being reduced in patients from the third month onwards, with most cases stabilizing at the six-month mark. In this period, opportunistic infection prevention treatment has usually finished, and a consistent, targeted therapy for comorbid conditions is typically applied for most patients.

This study has some limitations. A small group of paired surveillance biopsies was used to analyze TAC monitoring’s influence on histological graft lessons. Due to the COVID-19 pandemic, many patients, more than usual, did not agree to the performance of a surveillance biopsy. We did not have data about genetic variabilities and de novo donor-specific antibody development, which could have affected the course of kidney function and histological changes.

## 5. Conclusions

- The data in this study do not show an association between TAC exposure/metabolism and IF/TA progression during the first year after kidney transplantation. However, a higher TAC CV tertile was associated with a higher chronicity score at a one-year biopsy.
- Faster TAC metabolism was associated with reduced kidney graft function and an increased risk of rejection. Calculating the C/D ratio at three and six months after transplantation may help to identify patients at risk for acute rejection and deterioration of graft function and be a simple and inexpensive tool that is useful for physicians in their daily clinical practice. Based on this finding, we recommend considering more frequently monitoring fast tacrolimus metabolizers and a more cautious tapering of other immunosuppressive medicaments.
- Measuring tacrolimus blood level variability over six months to one year may help to identify patients at a greater risk of progression for chronic graft lesions and reduced long-term graft function. It may also point to potential non-adherence to immunosuppressive treatment.
- uNGAL, a possible marker of CNI-induced renal injury, negatively correlated with TAC C_0_ and C/D ratio at three months and one year and with IF/TA and chronicity scores at three-month biopsies.

There was no significant association between NGAL and KIM-1, tested the same time as a surveillance biopsy was performed, with rejection frequency and later graft function.

## Figures and Tables

**Table 1:** Donor and recipient (*n =* 174) characteristics.

Characteristics	Values
Donor age, years	51.39 ± 15.92
Donor type, SCD/ECD, (%)	87 (50.0)/87 (50.0)
Donor gender, male/female, (%)	97 (55.7)/77 (44.3)
Donor hypertension, %	52.9
Donor creatinine, µmol/L	81 (32–332)
Cold ischemia time, hours *	15.3 (9.0–34.3)
Recipient age, years	49.09 ± 12.88
Recipient gender male/female, (%)	104 (59.8)/70 (40.2)
Recipient hypertension, %	82.8
Diabetes mellitus, %	10.9
Recipient BMI, kg/m^2^	25.48 (18.2–38.9)
HLA mismatch *	3 (1–6)
Immunological risk, low/medium/high, (%)	7.0/80.2/12.8
DGF, %	26.4
Rejection during first year, %	13.8

BMI—body mass index; ECD—extended criteria donor; DGF—delayed graft function; SCD—standard criteria donor; *—values presented as median, min., or max.

**Table 2:** Tacrolimus monitoring data arranged according to interstitial fibrosis and tubular atrophy progression between biopsies.

Variable	No IF/TA Progression *n* = 59	IF/TA Progression *n* = 72	*p* Value
TAC dose/weight at 3 months (mg/kg)	0.121 ± 0.044	0.129 ± 0.48	0.453
TAC dose/weight at 1 year (mg/kg)	0.079 ± 0.046	0.085 ± 0.052	0.507
TAC C_0_ at 3 months (ng/mL)	9.14 ± 2.89	9.49 ± 2.96	0.49
TAC C_0_ at 6 months (ng/mL)	8.03 ± 2.4	7.59 ± 2.5	0.33
TAC C_0_ at 12 months (ng/mL)	7.53 ± 2.32	7.56 ± 2.48	0.945
C/D at 3 months (ng/mL/mg)	1.18 (0.32–5.73)	1.09 (0.28–5)	0.963
C/D at 6 months (ng/mL/mg)	1.28 (0.45–5.7)	1.33 (0.3–7.5)	0.235
C/D at 12 months (ng/mL/mg)	1.3 (0.52–4.7)	1.24 (0.19–5.95)	0.454
Fast metabolizer percentage at 6 months	46.3	39.7	0.457
Fast metabolizer percentage at 1 year	40.9	30.2	0.266
TAC-C_0_ CV, %	26.27 ± 11.49	27.88 ± 10.44	0.423
Third TAC-C_0_ tertile CV	40.07 ± 8.5	38.8 ± 6.6	0.678

C/D—concentration/dose ratio; CV—coefficient of variability; IF/TA—interstitial fibrosis and tubular atrophy; TAC—tacrolimus; TAC C_0_—tacrolimus trough concentration.

**Table 3:** Logistic regression analysis of tacrolimus monitoring and clinical data associations with first-year rejection.

Univariate				Multivariate		
	*OR*	95% CI	*p* Value	*OR*	95% CI	*p* Value
DGF	4.19	1.719–10.214	0.002	3.935	1.329–11.646	0.013
Recipient age, years	0.974	0.942–1.007	0.122			
Diabetes	3.513	1.187–10.396	0.023	3.882	1.150–13.112	0.029
Donor age, years	1.025	0.993–1.058	0.125			
Donor creatinine, µmol/L	1.007	0.999–1.014	0.083			
Cold ischemia time, hours	1.049	0.955–1.142	0.34			
TAC C_0_ at 6 months, ng/mL	0.78	0.622–0.977	0.031	0.752	0.570–0.991	0.043
Fast metabolizer at 3 months	3.017	1.214–7.497	0.017			
Fast metabolizer at 6 months	2.736	1.077–6.951	0.034			
Fast metabolizer at 1 year	2.30	0.888–5.958	0.081			
C/D at 3 months, ng/mL/mg	0.301	0.12–0.752	0.01	0.301	0.110–0.821	0.019
C/D at 6 months, ng/mL/mg	0.44	0.188–1.049	0.064			
C/D at 1 year, ng/mL/mg	0.444	0.205–0.962	0.04			

C/D—concentration/dose ratio; CI—confidence interval; DGF—delayed graft function; TAC C0— tacrolimus trough concentration.

**Table 4:** Univariate and multivariate logistic regression analysis for the predictors of graft function (eGFR < 60 mL/min/1.73 m^2^) at two years***.***

Univariate				Multivariate		
	*OR*	95% CI	*p* Value	*OR*	95% CI	*p* Value
Recipient age, years	1.001	0.974–1.029	0.933			
Donor age, years	1.033	1.009–1.056	0.006	1.043	1.001–1.086	0.045
Donor evaluation	3.175	1.484–6.790	0.003			
Donor hypertension	2.498	1.199–5.208	0.015			
Cold ischemia time, hours	1.117	1.013–1.232	0.027			
KRT time before transplantation, months	0.986	0.973–1.00	0.050			
TAC C_0_ at 6 months, ng/mL	0.832	0.707–0.979	0.027			
Fast metabolizer at 6 months	2.437	1.107–5.365	0.027	4.654	1.197–18.097	0.026
Rejection during first year	4.648	1.014–21.306	0.048			
IF/TA score at 1 year	2.185	1.212–3.939	0.009			
Chronicity score at 1 year	1.788	1.179–2.711	0.006	1.575	1.002–2.473	0.049

CI—confidence interval; IF/TA—interstitial fibrosis and tubular atrophy; KRT—renal replacement therapy; OR—odds ratio; TAC C_0_— tacrolimus trough concentration.

## Data Availability

The data are contained within the article and Supplementary Materials. The datasets used and analyzed during the current study are available from the corresponding author (M.R) on request The data are not publicly available due to [restrictions on their containing information that could compromise the privacy of research participants].

## Supplementary Materials

The following supporting information can be downloaded at: https://www.mdpi.com/article/10.3390/biomedicines12051125/s1, Table S1. Clinical data of patients according to interstitial fibrosis and tubular atrophy progression between surveillance biopsies; Table S2. Tacrolimus monitoring and biomarkers data according to interstitial fibrosis and tubular atrophy progression between biopsies.

## Abbreviations

BMI	body mass index
C/D	concentration doses ratio
CI	confidence interval
DGF	delayed graft function
ECD	expanded-criteria donor
eGFR	estimated glomerular filtration rate
HD	hemodialysis
IF/TA	interstitial fibrosis and tubular atrophy
KIM-1	kidney injury molecule-1
KRT	kidney replacement therapy
NGAL	neutrophil gelatinase-associated lipocalin
MMF	mycophenolate mofetil
OR	odds ratio
TAC CV	tacrolimus coefficient of variation
TAC C_0_	tacrolimus trough concentration.
