# Supplementary material for: The Influence of Tacrolimus Exposure and Metabolism on the Outcomes of Kidney Transplants
Source: Biomedicines. 2024 May 18;12(5):1125. doi: 10.3390/biomedicines12051125 (PMC11117915; doi:10.3390/biomedicines12051125)
Supplement: Supplementary file 1 [file biomedicines-12-01125-s001.zip › biomedicines-2990026-supplementary.pdf]

Supplement Table S1. Clinical data of patients according to interstitial fibrosis and tubular atrophy progression between surveillance biopsies.

**Variable**	**No IFTA progression**<br>**n**=59	**IFTA progression**<br>**n**=72	**P value**
Recipient age, years	50.51±12.02	44.31±13.13	**0.006**
Donor age, years	48.7±16.6	53.6±15/5	0.118
Donor evaluation SCD/ECD, %	59.3/40.7	43.1/56.9	0.085
Donor hypertension, %	37,3	63.9	**0.002**
Cold ischemia time, hours	16.27±4.84	16.22±4.3	0.651
DGF, %	16.9	27.8	0.142
Rejection episode, %	12.1	27.8	**0.028**
Proteinuria 1year g/l	0.107±0.24	0.124±0.37	0.704
eGFR at 3 months, ml/min/1,73m^2	57.14±16.28	47.82±16.45	**0.003**
eGFR at 6 months, ml/min/1,73m^2	54.14±19.75	44.99±19.79	**0.007**
eGFR at 1year, ml/min/1,73m^2	57.48±16.57	47.82±16.45	**0.002**
eGFR at 2 years, ml/min/1,73m^2	57.64±16.38	49.50±19.36	**0.033**
eGFR at 3 years, ml/min/1,73m^2	60.14±17.43	48.22±18.76	**0.012**

DGF-delayed graft function, ECD-extended criteria donor, eGFR-estimated glomerular filtration rate, IF/TA – interstitial fibrosis and tubular atrophy, SCD- standard criteria donor; values present as mean, percent

Supplement Table S2. Tacrolimus monitoring and biomarkers data according to interstitial fibrosis and tubular atrophy progression between biopsies.

Variable	No IF/TA progression from zero biopsy to 3months biopsy	IF/TA progression from zero biopsy to 3months biopsy	P value	No IF/TA progression from 3 months to 1-year biopsies	IF/TA progression from 3 months to 1-year biopsies	P value
TAC dosis/weight 3 months (mg/kg)	0.128±0.047	0.123±0.046	0.659	0.110±0.425	0.130±0.0479	0.142
TAC dosis/weight 6 months (mg/kg)				0.0911± 0.043	0.1120±0.060	0.260
TAC dosis/weight 12 months (mg/kg)				0.0765±0.379	0.0861±0.07	0.909
TAC C_0 3 months (ng/ml)	9.56±2.837	9.18±3.209	0.512	9.1059±3.029	9.831±2.244	0.076
TAC C_0 6 months (ng/ml)				7.9441±2.8194	7.0483±2.296	0.177
TAC C_0 12 months (ng/ml)				7.465±2.591	7.779±2.275	0.613
C/D 3 months (ng/ml/mg)	1.31±0.893	1.31±0.817	0.832	1.22±0.59	1.32±0.91	0.788
C/D 6 months (ng/ml/mg)				1.843±0.50	1.267±1.42	0.077
C/D 12 months (ng/ml/mg)				1.387±0.62	1.57±1.20	0.836
CV TAC-C_0	26.79±11.15	28.53±11.44	0.448	25.997±11.47	28.544±10.54	0.379
CV TAC-C_0 third tertile				39.975±7.28	38.052±6.172	0.52
Log sNGAL 3 months	3.943±0.414	4.038±0.568	0.418	4.066±0.44	3.71±0.43	**0.029**
Log sNGAL 12 months				3.91±0.37	4.086±0.45	0.671
Log uNGAL 3 months	2.997±0.715	3.3150±0.439	0.124	3.17±0.628	2.92±0.40	0.28
Log uNGAL 12 months				3.765±0.61	3.4788±0.83	0.328
Log uKIM-1 3 months	0.291±0.162	0.303±0.166	0.859	0.326±0.174	0.2878±0.014	0.586
Log uKIM-1 12 months	0.3107±0.245	0.2661±0.153	0.78	0.3107±0.245	0.2661±0.153	0.78
Fast metabolizer at 3 months %	43.2	52.4	0.343	50	48.8	0.682
Fast metabolizer at 6 months %				47.1	60.7	0.284
Fast metabolizer 1 year %				35.3	41.4	0.620

C/D-concentration doses ratio, CV-coefficient of variability, IF/TA – interstitial fibrosis and tubular atrophy, uKIM-1-urine kidney injury molecule-1, NGAL- serum and urine neutrophil gelatinase–associated lipocalin, TAC-tacrolimus, TAC C_0- tacrolimus trough concentration;

values present as mean, percent;

biomarkers are log transformed
